# Superficial Temporal Artery and Vein as Alternative Recipient Vessels for Intraoral Reconstruction With Free Flaps to Avoid the Cervical Approach With the Resulting Need for Double Flap Transfer in Previously Treated Necks

**DOI:** 10.3389/fonc.2022.879086

**Published:** 2022-07-07

**Authors:** Lucas M. Ritschl, Minli Niu, Katharina Pippich, Philia Schuh, Niklas Rommel, Andreas M. Fichter, Klaus-Dietrich Wolff, Jochen Weitz

**Affiliations:** ^1^ Department of Oral and Maxillofacial Surgery, Klinikum rechts der Isar, School of Medicine, Technical University of Munich, Munich, Germany; ^2^ Department of Oral and Maxillofacial Surgery, Josefinum, Augsburg and Private Practice Oral and Maxillofacial Surgery im Pferseepark, Augsburg, Germany

**Keywords:** intraoral reconstruction, temporal vessel, microvascular anastomosis, osteoradionecrosis, free fibula flap

## Abstract

**Background:**

Microvascular reconstruction remains challenging in previously operated and irradiated patients, especially when double flaps seem to be the only solution due to osteoradionecrosis. An alternative reconstructive option could be microvascular anastomosis to the temporal vessels to avoid the obligatory cervical incision.

**Methods and Materials:**

All consecutive cases between January 2013 and 2020 that underwent either mandibular resection and reconstruction with a free fibula flap (FFF) and another soft tissue flap (group I) or pure intraoral resection and reconstruction with an FFF or radial forearm flap (RFF) with temporal microvascular anastomosis (group II) were included. Patients’ general information, indication and extent of surgery, time of ischemia, time of total surgery, and duration of hospital stay as well as incidence of complications were retrospectively recorded and analyzed.

**Results:**

Seventeen (group I) and 11 (group II) cases were included. In group I, FFF was combined with RFF (*n* = 9), anterolateral thigh flap (ALT, *n* = 7), or latissimus dorsi flap (*n* = 1). Group II consisted of six FFFs and five RFFs. Operation time and hospitalization duration were significantly shorter in group II (*p *< 0.001 and *p* = 0.025), whereas ischemic time of FFF was significantly shorter in group I (*p* = 0.002). All patients in group I required a tracheostomy, while only four cases in group II did (*p* = 0.004). The complication rate regarding hematoma removal, revision of anastomosis, flap loss, delirium, sepsis, pleural effusion, pneumonia, and pulmonary artery embolism showed no significant differences between the two groups.

**Conclusions:**

The superficial temporal vessels served as versatile recipient vessels for intraoral mandibular and soft tissue reconstruction and led to reduced operation time, hospitalization duration, and indication for a primary tracheostomy. Thus, this approach may help to avoid cervical incision for reconstruction in irradiated patients.

## Introduction

Free flap reconstruction and mandibular reconstruction in previously operated and irradiated head and neck regions remain challenging and are more often associated with complications (1–3). Furthermore, osteoradionecrosis (ORN) of the jaws represents a common and severe complication after primary or adjuvant radiation therapy (RT) of large head and neck tumors. Osteoradionecrosis is reported in 5%–15% of irradiated cases and mainly evolves in the first few years after the end of treatment (4–6). Furthermore, ORN is significantly more prevalent in the mandibular bone than in the maxilla at a ratio of approximately 24:1 because of the relatively poor vascularization and higher density of the bone (7–11). In advanced ORN cases, surgical management is generally considered the therapy of choice. However, in several severe ORN patients with extensive bone and soft tissue defects with or without intra-extraoral fistulation, functional and esthetic reconstruction represents a huge challenge for the patients and the whole team. An increased risk of postoperative wound-healing disorders—especially on the neck site due to altered immune competence—contributes to prolonged hospitalization duration and reduced quality of life (1, 12).

Various strategies are described to avoid these complications and to solve this clinically demanding situation (13). These strategies include a revival of pedicled local flaps, harvest of a chimeric free fibula flap (two fasciocutaneous paddles based on a second random perforator or an additional soleus perforator flap) (14–16) or intraoral lining with the muscles (posterior tibial and flexor hallucis longus muscles), and extraoral positioning of the fasciocutaneous flap. Another more standardized and widely performed approach is the transfer of double or sandwich flaps (11, 17, 18).. Accordingly, double flaps need more cervical recipient vessels and a third surgical team harvesting the second free flap simultaneously. Alternative solutions—in terms of reduction of extraoral invasiveness—are rare but required, especially if the cervical vessel situation may not allow double flap reconstruction due to multiple surgeries in the neck region. One solution could be the pure intraoral resection and microvascular anastomoses to the facial artery and vein as described by others (19, 20). However, this approach is not feasible in patients with a history of (multiple) cervical operations—like neck dissection in oncologic cases—resulting in a vessel-depleted neck situation.

In this sense, the superficial temporal artery and vein (STA/V) could be promising recipient vessels for intraoral free flap reconstruction. The STA/V are frequently used for extraoral reconstruction of the upper two-thirds of the face and are the gold-standard recipient vessels for scalp reconstruction because of their reliable anatomical course and suitable diameter for most free flaps (21–24).

The purpose of this study is to compare both strategies—double flap transfer and pure intraoral resection with temporal microvascular anastomoses for intraoral reconstructions—regarding feasibility, operation time, and complication rates in these clinically challenging reconstructive cases as the choice of recipient vessels and options available to the reconstructive surgeon in case of retreatment with a free flap is limited.

## Materials and Methods

### Ethical Statement and Enrolled Patients

All clinical investigations were conducted according to the principles expressed in the Declaration of Helsinki. The retrospective study was approved by the Institutional Ethics Committee of the Technical University of Munich, Klinikum rechts der Isar (Approval number: 424/19 S-SR).

The study cohort consisted of two groups: (I) double flap and (II) temporal microvascular anastomosis. All patients who underwent either mandibular resection and reconstruction with a free fibula flap (FFF) and another soft tissue flap (group I) or intraoral reconstruction with an FFF or radial forearm flap (RFF) with microvascular anastomoses to the STA/V (group II) between January 2013 and 2020 were included.

Data collection included gender, age, indication for mandibular reconstruction, extent of resection according to Brown et al. (I–IV) (25), number of fibular segments, duration of surgery (min), ischemia time (min), duration of stay on the intermediate care unit (ICU) (days), duration of tracheostoma (days), and hospital stay (days). Ischemia time was defined as the interval between ligation of the pedicle and opening of the vessel clamps following microvascular anastomoses. In cases of reconstruction with FFF in group II, ischemia time also included mandibular reconstruction with completed osteosynthesis.

### Preoperative Management and Procedures in Group II

In the case of the double flap procedure, each patient received a computed tomography angiography (CTA) to visualize the supra-aortic and lower leg vessels. A three-team approach was necessary to reduce the operation time. In the case of a three-vessel supply of the lower leg, the harvest of an FFF was further planned for bony mandibular reconstruction. Care was taken to ensure that the osteomyocutaneous island of the FFF was positioned intraorally to avoid under- or overcrossing the FFF bone of the second flap. This essential planning actively avoided potential functional stenosis and/or kinking of the second flap’s pedicle. Consequently, the second flap was positioned extraorally, and an RFF, an anterolateral thigh flap (ALT), or a latissimus dorsi flap was applied according to the defect size of the cervical region to achieve a tension-free wound closure.

### Preoperative Management, Surgical Approach, and Procedures in Group II

**Figure 1:**
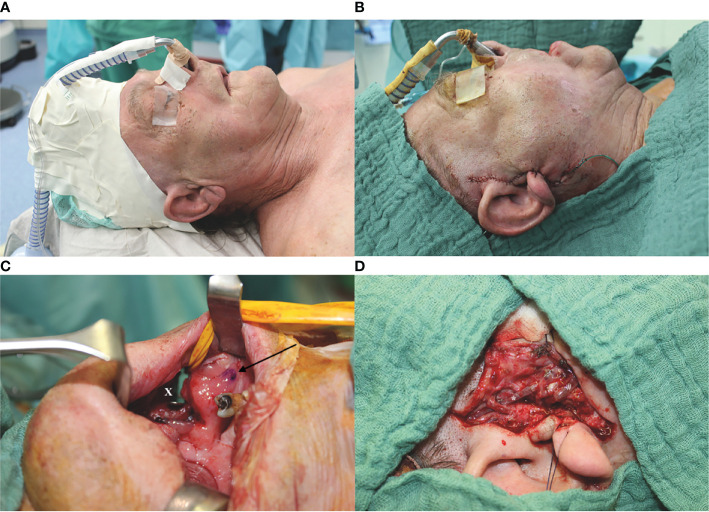
Exemplary case demonstration with temporally anastomosed one-segmented osteomyocutaneous fibula free flap because of osteoradionecrosis of the right mandibular body. Preoperative CT angiography revealed no reasonable cervical external carotid branches for microvascular anastomosis due to previous operations: **(A)** preoperative situation; **(B)** postoperative situation without cervical scars after mandibular reconstruction; **(C)** preparation of the transfacial subcutaneous tunnel (x) with careful preservation of the parotid duct, its intraoral ostium (arrow), and the accompanying buccal branch of the facial nerve; and **(D)** temporal anastomoses with the superficial temporal artery and vein (STA/V). The STV has been divided to allow the anastomoses of two veins, one draining retrograde to the superficial scalp (*) and the other orthograde into the deep cervical venous system (#).

If the temporal vessels were present, this option was considered and discussed with the patient. The surgeon then palpated the temporal artery and used a handheld Doppler (Handydop^®^, ELCAT GmbH; Wolfratshausen, Germany) to verify the CTA. Intraoperatively, preauricular incision was performed according to the conventional preauricular approach. Subsequently, the STA/V were located cranially of the zygomatic arch, circumferentially freed, and prepared as the recipient vessels. For this purpose, the STV was divided 15–20 mm cranial to the zygomatic arch, resulting in two recipient veins, one draining retrograde to the superficial scalp system and the other orthograde into the deep cervical vein system. Thereafter, the better draining pedicle’s vein was anastomosed to the deep cervical system. The preparation of the transfacial subcutaneous tunnel then began. The superficial musculoaponeurotic system (SMAS) was identified and successively bluntly dissected solely above the SMAS until the anterior border of the masseteric muscle was reached. After bi-digital palpation, the correct localization of the intraoral incision was defined under careful preservation of the parotid duct, its intraoral ostium, and the accompanying buccal branch of the facial nerve. Extra- and intraoral tunnel preparation was bluntly united with adequate width for the pedicle, as generally known from cervical preparations with a meticulous hemostasis (**Figure 1**).

**Figure 2:**
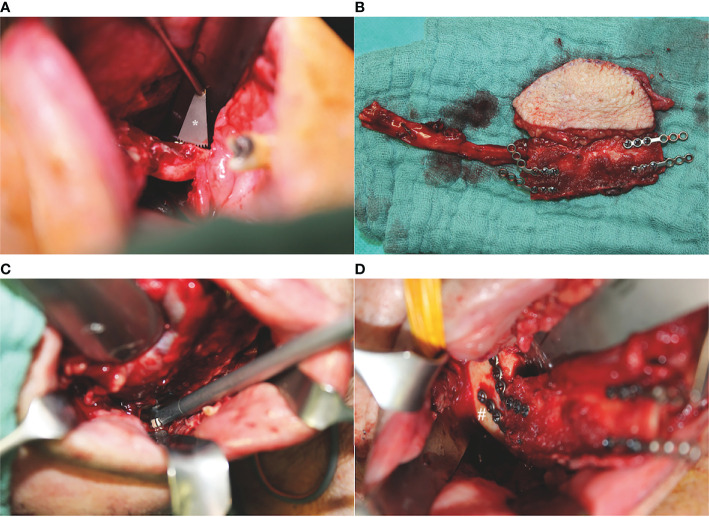
Intraoperative situations with angulated instruments for pure intraoral mandibular resection and osteosynthesis of a one-segmented osteomyocutaneous fibula free flap: **(A)** mandibular osteotomy with an angulated microsaw (*) with an integrated rinsing system (Medicon; Tuttlingen, Germany), **(B)** one-segmented osteomyocutaneous fibula free flap with pre-bent 2.0 mini-plates, **(C)** 90° angulated drill and screwdriver (Modus^®^ 2 90° Luhr Fritzmeier screwdriver; Medartis AG; Basel, Switzerland) for osteosynthesis at the right mandibular angle, and **(D)** intraoperative reconstructive result after completed osteosynthesis with monocortical screws at the right mandibular angle (#).

Intraoral mandibular resection was performed applying an angulated microsaw with an integrated rinsing system (Medicon; Tuttlingen, Germany) and using CAD/CAM cutting guides. The FFF was positioned intraorally with the vascular pedicle running orally to the fibular bone. At that point, kinking of the vascular pedicle must be ruled out and the intraoral incision of the tunnel may be adapted. Following this, 2.0 mini-plates with monocortical screws were used for osteosynthesis using a 90° angulated drill and screwdriver (Modus^®^ 2 90° Luhr Fritzmeier screwdriver; Medartis AG; Basel, Switzerland) (**Figure 2**). Finally, the pedicle was guided carefully through the prepared transfacial tunnel to the temporal recipient vessels, and conventional microvascular anastomosis was performed.

### Statistical Analysis

For the non-normally distributed results, the median (range) was presented. For the statistical comparison of the two groups, a Mann–Whitney *U* test with an exploratory two-sided 5% significance level was performed. Uni- and multivariate regression analyses of possible confounders on hospitalization duration were performed.

No adjustments were made for multiple testing. Analysis was done with IBM SPSS 24 for Mac software (IBM Corp., Armonk, NY, USA).

## Results

### General Information and Descriptive Statistics

General information regarding the distribution of age, gender, BMI, ASA status, indication, and mandibular defect of both groups is listed in **Table 1**. A positive history of radiation prior to reconstructive surgery at our department was recorded in 100% of patients in group I and 90.9% in group II. Previous surgical procedures at the neck had been conducted in 82.4% in group I and in 81.8% in group II. The following additional free flap combinations with FFF were included in the double flap group (I): FFF + RFF (*n* = 9), FFF + ALT (*n* = 7), and FFF + latissimus dorsi flap (*n* = 1). In the temporal anastomosis group (II), six FFFs and five RFFs were included.

**Table 1:** Patient records and specifications of both groups [double flap (*n* = 17) and temporal anastomosis (*n* = 11)].

Parameter	Double flap		Temporal anastomosis		*p*-value^a^
Age	58 (47–76)		70 (49–78)		0.005
Gender	4 female/13 male		4 female/7 male		0.578
BMI	21.4 (13.4–27.3)		23.8 (18.4–36.3)		0.033
ASA status	I II III IV	0 14 3 0	I II III IV	0 7 4 0	0.430
Indication	OSCC ORN WHD 2° recon.	0 12 0 5	OSCC ORN WHD 2° recon.	1 6 4 0	0.643
Mandibular defect (Brown et al.)	I II III IV	5 3 0 9	I II III IV	2 1 0 3	0.649

BMI, body mass index [kg/m (2)]; ASA, American Society of Anesthesiologists; 2° recon., secondary reconstruction. ^a^Mann–Whitney U test with an exploratory two-sided 5% significance level.

**Figure 3:**
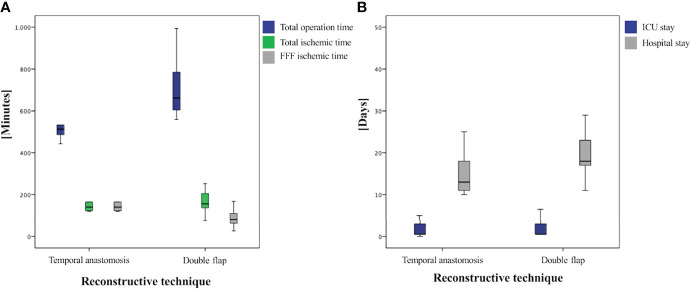
Boxplots of analyzed times: **(A)** comparison of operation-specific times and **(B)** comparison of ICU and total hospital stay.

Operation time was significantly shorter in group II [486.0 min (378.0–646.0)] than in group I [662.0 min (559.0–994.0); *p *< 0.001; **Table 2** and **Figure 3**]. The ischemic time of FFF in group I (81.0 min (26.0–168.0)] was significantly shorter than in group II [140.5 min (120.0–248.0); *p* = 0.002; **Table 2** and **Figure 3**].

**Table 2:** Specifications of the operation and hospital stay [incidence or median (range)] of both groups [double flap (*n* = 17) and temporal anastomosis (*n* = 11)].

Parameter	Double flap		Temporal anastomosis		*p*-value^a^
Operation time (min)	662.0 (559.0–994.0)		486.0 (378.0–646.0)		<0.001
Total ischemic time (min)	156.0 (76.0–322.0)		120.0 (58.0–248.0)		0.013
FFF ischemic time (min)	81.0 (26.0–168.0)		140.5 (120.0–248.0)		0.002
Primary tracheotomy	17 (100%)		4 (36.4%)		0.004
Removal of tracheostoma (days)	6.0 (2.0–41.0)		3.0 (3.0–8.0)		0.101
PEG	No need	4	No need	5	0.378
	Present	7	Present	3	
	New	6	New	2	
	Change	0	Change	1	
ICU stay [day]	0.5 (0.5–33.0)		0.5 (0.0–11.0)		0.611
Hospital stay [day]	18.0 (11.0–49.0)		13.0 (10.0–25.0)		0.025

FFF, fibula free flap; PEG, percutaneous endoscopic gastrostomy; ICU, intensive care unit. ^a^Mann–Whitney U test with an exploratory two-sided 5% significance level.

All patients in group I required a tracheostomy, which was only necessary in four cases in group II (36.4%; *p* = 0.004). A secondary tracheostomy was never indicated in group II. A return from the normal ward to the ICU due to delirium was indicated twice in the double flap group (11.8%) and once in the temporal anastomosis (9.1%) group (*p* = 0.926).

The duration of stay in the ICU was in median of the regularly planned 0.5 days in both groups and showed no significant difference (*p* = 0.611), whereas total hospital stay was significantly shorter in group II at 13.0 days (10.0–25.0) than in group I at 18.0 days (11.0–49.0; *p* = 0.025; **Table 2** and **Figure 3**). A closer descriptive comparison of group I is shown in **Table 3**. All analyzed times were longer in the FFF temporal anastomosis group. **Table 4** displays a descriptive bone segment-related comparison between group I and the FFF reconstructions of group II.

**Table 3:** Comparison between radial forearm (*n* = 5) and free fibula (*n* = 6) flaps in the group of temporal anastomosis (group II) [incidence or median (range)].

Parameter	RFF	FFF
Operation time (min)	387.0 (378.0–518.0)	513.0 (443.0–646.0)
Total ischemic time (min)	76.0 (58.0–118.0)	140.5 (120.0–248.0)
Primary tracheotomy	2	2
Removal of tracheostoma (days)	3.0 (3.0–8.0)	3.0
ICU stay (days)	0.5 (0.0–0.5)	2.0 (0.5–11.0)
Hospital stay (days)	11.0 (10.0–16.0)	18.0 (11.0–25.0)

RFF, radial forearm flap; FFF, fibula free flap; PEG, percutaneous endoscopic gastrostomy; ICU, intensive care unit.

**Table 4:** Comparison of mandibular reconstructions with the fibula free flap in both groups: double flap (= I) versus temporal anastomosis (= II).

Group and number of segments	Operation time (min)	FFF ischemic time (min)	Primary tracheotomy (*n*)	Removal of tracheostoma (days)	ICU stay (days)	Hospital stay (days)
I 1-segmented FFF (*n* = 3)	704.0 (604.0–744.0)	81.0 (68.0–113.0)	3	6.0 (4.0–13.0)	0.5 (0.5–0.5)	17.0 (17.0–25.0)
II 1-segmented FFF (*n* = 4)	508.0 (443.0–533.0)	125.5 (120.0–248.0)	1	3.0	3.0 (0.5–11.0)	15.5 (11.0–25.0)
I 2-segmented FFF (*n* = 6)	661.0 (595.0–832.0)	90.5 (50.0–167.0)	6	3.0 (2.0–41.0)	0.5 (0.5–33.0)	17.5 (11.0–49.0)
II 2-segmented FFF (*n* = 2)	571.0 (496.0–646.0)	159.0 (153.0–165.0)	1	3.0	2.0 (1.0–3.0)	19.0 (18.0–20.0)
I 3-segmented FFF (*n* = 8)	641.0 (559.0–994.0)	77.5 (26.0–168.0)	8	14.0 (3.0–41.0)	0.5 (05.–5.0)	21.5 (15.0–43.0)
II 3-segmented FFF (*n* = 0)	/	/	/	/	/	/

Median (range). group I, double flap group; group II, temporal anastomosis group; FFF, fibula free flap; ICU, intensive care unit.

Complication rate regarding hematoma removal, revision of anastomosis, flap loss, delirium, sepsis, pleural effusion, pneumonia, and pulmonary artery embolism showed no significant difference in incidence between the two groups (**Table 5**). Temporary or permanent impairment of the facial nerve branches (especially marginal mandibular and buccal) was not registered in both groups.

**Table 5:** Group-related complications: double flap versus temporal anastomosis. .

Parameter		Double flap	Temporal anastomosis	*p*-value^a^
Surgical nature	Total (*n*)	7	5	0.904
	Hematoma removal	1 (5.9%)	2 (18.2%)	0.313
	Revision anastomosis	4 (23.5%)	2 (18.2%)	0.741
	Flap loss	2 (11.8%)	1 (9.1%)	0.826
Mental nature	Delirium (*n*)	3 (17.6%)	0 (0.0%)	0.148
Internistic nature	Total (*n*)	8	3	0.972
	Sepsis	1 (5.9%)	0 (0.0%)	0.421
	Pleural effusion	3 (17.6%)	2 (18.2%)	0.972
	Pneumonia	3 (17.6%)	1 (9.1%)	0.535
	Pulmonary artery embolism	1 (5.9%)	0 (0.0%)	0.421

a Mann–Whitney U test with an exploratory two-sided 5% significance level.

### Uni- and Multivariate Linear Regression Analyses

The results of uni- and multivariate linear regression analyses of possible confounders on the duration of hospital stay are shown in **Table 6**. Reconstruction technique, operation time, total ICU time, and removal of tracheostoma showed a significant influence on the duration of hospital stay in the univariate linear regression analysis. Only total ICU time had a significant influence in the multivariate linear regression analysis.

**Table 6:** Uni- and multivariate regression analyses of possible confounders on hospital stay. .

Parameter	*p*-value	95% confidence interval
Univariate regression analysis—hospital stay		
Reconstruction technique	0.042	0.287 to 13.713
Operation time	0.031	0.002 to 0.045
1° tracheostomy	0.167	−2.435 to 13.387
Total time ICU	<0.001	0.543 to 1.345
Removal of tracheostoma	0.001	0.218 to 0.757
Delirium	0.830	−10.275 to 12.701
Multivariate regression analysis—hospital stay		
Reconstruction technique	0.508	−6.177 to 11.999
Operation time	0.946	−0.28 to 0.027
Total time ICU	0.013	0.172 to 1.263
Removal of tracheostoma	0.103	−0.055 to 0.541

ICU, intensive care unit.

## Discussion

This study investigated two very complex reconstructive options in a highly challenging patient population. In particular, the combination of a preoperated and irradiated neck reduces the reconstruction options to free microvascular flaps and may jeopardize the success rate of free tissue transfer as well as increase the complication rates (1, 26). Additionally, it is known that once the skin of the neck of such patients is incised, often another free flap is necessary for tension-free cervical wound closure because of the radiogenic dermatofibrosis, altered extracellular matrix remodeling, and changes in vasculature and local host defense peptides, which significantly reduce the natural cervical cutaneous laxity and healing capacity (12, 27).

This study collective included patients who had previously undergone surgery and/or radiation therapy and presented to our department primarily because of mandibular destruction on the underlying reason for osteoradionecrosis with intra- and/or extraoral fistulation or persistent wound-healing disturbance. With this anamnestic history and clinical features, a double flap approach would have been the most widely accepted solution. However, with the presented study, we revisited the temporal vessels as good and reliable alternative recipient vessels for intraoral microvascular reconstruction because of two reasons: limited cervical recipient vessels and prevention of cervical incision. In this context, we first describe a clinical case series with intraoral soft tissue or mandibular resection and consecutive reconstruction with an osteomyocutaneous FFF or soft tissue free flap with microvascular anastomoses to the STA/V. This approach for resection and reconstruction is a combination of previously described methods to reduce cervical invasiveness. Pure intraoral mandibular resection has been previously described, especially for benign tumors, to reduce extraoral scars and to preserve facial and cervical appearance (28). In contrast to our pure intraoral mandibular resections in group II, others have performed in some situations an additional temporal or preauricular incision, for example in cases with ramus involvement (19, 29). Garcia-Diez et al. reported in a case series about seven lateral mandibular defects that had been reconstructed with six iliac crest flaps and one FFF (29). In this case series, only benign tumors were resected. Furthermore, the STA/V were used as the recipient vessels only in two of the seven cases for the microvascular anastomoses. In our study, intraoral anastomoses to the facial vein and artery as previously described by others (28, 30) were not possible because of previous neck dissection and consecutive ligation of the facial vessels. Furthermore, the lingual pedicle position was associated with a consequent loss of visual control because of the intraoral fasciocutaneous island of the FFF for intraoral coverage. In benign bony processes of the mandible, a fasciocutaneous island of the FFF is not needed in most of the cases or a microvascular iliac crest flap is used. This fact increases the visual control and would make intraoral anastomosis significantly more feasible.

Some advantages of the applied reconstructive approach in group II over the double flaps became evident in this comparison. First, with the use of STA/V as the recipient vessels, only one microsurgical flap was needed, which significantly reduced the required manpower and time of surgery, as this procedure was undertaken in a conventional two-team approach. However, regarding manpower, two other modifications could have also dismissed the need for a second flap if one had gone the conventional transcervical approach: 1) harvest of a chimeric FFF (two fasciocutaneous paddles based on a second random perforator or an additional soleus perforator flap) (14–16) or 2) intraoral lining with the muscles (posterior tibial and flexor hallucis longus muscles) and extraoral positioning of the fasciocutaneous flap. Both options represent feasible solutions but have also only been described in case series in the literature, lacking comparison to the scientifically more standardized procedure as the double or sandwich flap. For this reason, a comparison of the temporally anastomosed intraoral reconstructions with that of the double flap group was made in this study. Second, the duration of hospital stay could be significantly reduced. This might positively correlate with the reduced time of surgery (31), but also with the reduced number of donor sites, which consequently minimizes the risk of pain, patient discomfort, and wound-healing disturbance. The minimized risk of swelling intraorally and in the neck region may also have led to a significantly lower rate of primary tracheostomy among the patients in group II (36.4% vs. 100%, *p* = 0.004, **Table 2**), which also could have influenced the hospitalization duration positively, as reported by others (32). Even though tracheostomy is an important procedure in upper airway management in microvascular flap transfer, it may be associated with several (severe) complications such as pneumonia, hemorrhage, stenosis/stricture, malacia, rupture, and *via* falsa (33–35). The risk may be further aggravated in cases of retracheostomy. However, as mentioned by others, mandibular reconstruction without primary tracheostomy should only be performed in a selected patient collective (36). Resection of the anterior symphysis segment with a consequent detachment of the supraomohyoidal muscles, including the anterior belly of digastric, geniohyoidal, and genioglossal muscles, as seen in Brown classes II–IV, will mostly lead to destabilization and retrusion of the floor of the mouth and indicate therefore a tracheostomy, even though these muscles are reattached to the neomandibula.

A significant disadvantage of the described procedure is the quite complex intraoral bony reconstruction, in which the most meticulous attention must be paid to the tension-free and straightest possible course of the vascular pedicle without any kinking. This is also reflected in the significantly longer ischemia time of FFF compared to group I and generally longer operation and ischemia time in comparison to intraoral reconstruction with RFF within group II (**Tables 2** **–** **4**). For this reason, intraoral resection and vessel preparation as well as flap raising and reconstruction were performed by senior consultants of our clinic. Nevertheless, the influence of surgical experience by varying surgeons cannot be ruled out in this university hospital setting as well as in this retrospective study (37, 38). According to recent CUSUM analyses by Han et al. and Zhu et al., the learning curve in microsurgical reconstruction or mandibular reconstruction stabilizes after 20 or 8–17 cases (38, 39). However, the bony reconstruction was always well manageable by the operating senior consultant with the use of angled tools and pre-bent mini-osteosynthesis plates. Another restricting aspect of this method is the need for a sufficiently long vascular pedicle of at least approximately 8 cm, as the distance from the mandibular angle or ascending ramus to the preauricular region must be bridged tension-free. Thus, this reconstructive option may be limited in the case of caudally outgoing fibular vessels—as the peroneal artery branching and the length of the tibial-peroneal trunk are associated with known variation (40, 41)—or LCL three-segment reconstruction, or requires the use of interposition vein grafts in these cases.

Despite the mentioned and manageable drawbacks, this approach should be included in the repertoire of soft tissue intraoral and mandibular reconstruction with a free flap next to chimeric FFF and muscular intraoral lining with the FFF. These motioned approaches represent new reconstructive solutions for patients with a history of radiotherapy in the head and neck region, neck dissections, and consecutive clinical situations of a radiogenic dermatofibrosis or with a vessel-depleted neck, which would traditionally indicate a double flap reconstruction.

### Limitations

The group sizes were too small and heterogeneous to allow final statements on whether this method also leads to fewer general complications such as pleural effusion, pneumonia, and pulmonary artery embolisms. However, this comparison already showed that the incidence of postoperative pneumonia or pulmonary artery embolism was reduced (**Table 5**), but not significantly. Only one alternative surgical approach/procedure was compared to the standardized double flap reconstruction on multiply operated and irradiated patients. A comparison to other mentioned options would be interesting, especially in a prospective setting with special regard to flap survival, operation and hospital stay times, incidence of complications, and function.

## Conclusion

The STA/V served as versatile recipient vessels for intraoral mandibular and soft tissue reconstruction in a challenging cohort. This reconstructive approach led to reduced operation time, duration of hospital stay, and indication for a primary tracheostomy in comparison to the double flap technique. Thus, this approach may help to avoid cervical incision for reconstruction in irradiated patients, which might indicate a second flap for cervical wound closure in this demanding patient population.

## Data Availability Statement

The original contributions presented in the study are included in the article/supplementary material. Further inquiries can be directed to the corresponding author.

## Ethics Statement

The studies involving human participants were reviewed and approved by institutional ethics committee of the Technical University of Munich, Klinikum rechts der Isar (Approval number: 424/19 S-SR). Written informed consent for participation was not required for this study in accordance with the national legislation and the institutional requirements.

## Author Contributions

LR: study design/conduction, operations, data interpretation, and major contribution to manuscript writing and revision. MN: data acquisition and interpretation, statistical analysis, and contribution to revision. KP: data interpretation, figure design, and contribution to revision. PS: data acquisition and interpretation, statistical analysis, and contribution to revision. NR: creation of figures and contribution to revision. AF: data interpretation, operations, and contribution to revision. K-DW: data interpretation, operations, and contribution to revision. JW: study design/conduction, data interpretation, and major contribution to manuscript writing and revision. All authors contributed to the article and approved the submitted version.

## Conflict of Interest

The authors declare that the research was conducted in the absence of any commercial or financial relationships that could be construed as a potential conflict of interest.

## Publisher’s Note

All claims expressed in this article are solely those of the authors and do not necessarily represent those of their affiliated organizations, or those of the publisher, the editors and the reviewers. Any product that may be evaluated in this article, or claim that may be made by its manufacturer, is not guaranteed or endorsed by the publisher.
